# Target of Rapamycin Signaling Involved in the Regulation of Photosynthesis and Cellular Metabolism in *Chlorella sorokiniana*

**DOI:** 10.3390/ijms23137451

**Published:** 2022-07-04

**Authors:** Linxuan Li, Tingting Zhu, Lele Huang, Maozhi Ren

**Affiliations:** Institute of Urban Agriculture, Chinese Academy of Agricultural Sciences, Chengdu National Agricultural Science and Technology Center, Chengdu 610000, China; lilinxuan@caas.cn (L.L.); zhutingting@caas.cn (T.Z.); huanglele63@outlook.com (L.H.)

**Keywords:** target of rapamycin, AZD8055, photosynthesis, *Chlorella sorokiniana*

## Abstract

Target of rapamycin (TOR) is a serine/threonine protein kinase that plays a central regulating role in cell proliferation, growth, and metabolism, but little is known about the TOR signaling pathway in *Chlorella sorokiniana*. In this study, a *Chlorella sorokiniana* DP-1 strain was isolated and identified, and its nutritional compositions were analyzed. Based on homologous sequence analysis, the conserved CsTOR protein was found in the genome of *Chlorella sorokiniana*. In addition, the key components of TOR complex 1 (TORC1) were present, but the components of TORC2 (RICTOR and SIN1) were absent in *Chlorella sorokiniana*. Pharmacological assays showed that *Chlorella sorokiniana* DP-1 was insensitive to rapamycin, Torin1 and KU0063794, whereas AZD8055 could significantly inhibit the growth of *Chlorella sorokiniana*. RNA-seq analysis showed that CsTOR regulated various metabolic processes and signal transduction pathways in AZD8055-treated *Chlorella sorokiniana* DP-1. Most genes involved in photosynthesis and carbon fixation in *Chlorella sorokiniana* DP-1 were significantly downregulated under CsTOR inhibition, indicating that CsTOR positively regulated the photosynthesis in *Chlorella sorokiniana*. Furthermore, CsTOR controlled protein synthesis and degradation by positively regulating ribosome synthesis and negatively regulating autophagy. These observations suggested that CsTOR plays an important role in photosynthesis and cellular metabolism, and provide new insights into the function of CsTOR in *Chlorella sorokiniana*.

## 1. Introduction

Microalgae is the general term for microorganisms that contain chlorophyll and can perform photosynthesis, and is an important component of the earth’s ecosystem; half of carbon dioxide fixation is completed by algae globally [1]. Microalgae is not only distributed in various water bodies, but also found in extreme environments such as deserts, hot springs and glaciers. *Chlorella sorokiniana*, a kind of unicellular green alga, has the advantages of fast growth, high lipid content and strong adaptability to various extreme environments or culture conditions. *Chlorella sorokiniana* can be used for large-scale cultivation and production of biomass, oil and other bulk raw materials, and has important industrial value in biofuel production [2]. At present, there are many reports on increasing the biomass and oil production of microalgae. Studies have found that efficient algal biomass and oil production can be achieved by a new culture strategy of heterotrophy–dilution–photoinduction [3]. In addition, mixed culture conditions with simultaneous addition of sugar and light can also stimulate efficient oil production. Recent studies have shown that most proteins in *Chlorella sorokiniana* are highly expressed under a light intensity of 70–110 μmol m^−2^ s^−1^, so it is expected to achieve high-density fermentation and high biodiesel production by adjusting the light intensity [4]. *Chlorella sorokiniana* grows rapidly, has a high biological yield under heterotrophic conditions and is considered to be the preferred strain for in-depth study on heterotrophic cell oil production. Among them, *Chlorella sorokiniana* UTEX 1230 is one of the strains identified with the highest production [5]. In a bioreactor with optimal culture conditions, *Chlorella sorokiniana* UTEX 1230 could accumulate 30–40% of its cell mass in the form of lipids [6]. A study found that the total lipid (dry weight) accumulated during the heterotrophic growth of *Chlorella sorokiniana* UTEX 1230 was up to 39%, whereas the total lipid accumulated during photoautotrophic growth was only 18% [6].

Target of rapamycin (TOR) is an evolutionarily conserved serine/threonine protein kinase that can regulate transcription, protein translation, cellular metabolism and other molecular activities by integrating nutrient, energy and growth factors, and plays a central regulating role in cell proliferation, growth and metabolism processes [7,8,9,10]. As the master regulator of nutrient, energy and stress signaling networks, TOR kinase is highly conserved in the evolution of all eukaryotes from yeast, plants and animals, to humans [11,12]. As a TOR-specific inhibitor, rapamycin is widely used in studies on TOR function [13]. The inhibition of TOR activity by rapamycin requires the mediation of FKBP12. After binding to FKBP12, the rapamycin–FKBP12 complex targets the FRB domain of TOR to form a ternary complex that prevents the binding of TOR and RAPTOR, thus inhibiting the activity of TOR kinase [14]. Studies on the TOR signaling pathway in plants lag behind those on yeast and mammals because most terrestrial plants are insensitive to rapamycin and TOR mutation leads to embryonic death, which hinders the study of plant TOR function [15]. It is worth noting that *Chlamydomonas reinhardtii*, a green alga, contains conserved TOR protein and is sensitive to rapamycin. In the presence of rapamycin, CrTOR binds to CrFKBP12 and inhibits CrTOR activity, thus preventing the cell growth of *Chlamydomonas reinhardtii* [16]. TOR can also affect biomass accumulation and cell cycle progression by synchronously changing the carbon and nitrogen balance of *Chlamydomonas reinhardtii* cells [17]. Studies have found that enhanced CO2 fixation could improve TOR activity, whereas inhibition of Calvin–Benson–Bassham (CBB) cycle and photosynthesis could decrease TOR activity in *Chlamydomonas reinhardtii* [18]. The application of novel active site inhibitors of TOR (asTORis) partially overcomes the problem of plants and algae being insensitive to rapamycin [13,19,20]. Among these asTORis, Torin1, KU0063794, PP242 and AZD8055 have been widely used in the studies on plant TOR function [20,21,22,23,24]. Recent studies have found that TOR inhibitors can induce the accumulation of triacylglycerols (TAGs) in the cells of *Cyanidioschyzon merolae* and *Chlamydomonas reinhardtii* [19]. A recent study on *Euglena gracilis*, an ancient alga belonging to Excavata, showed that the TOR inhibitor rapamycin had less effect on the cell proliferation of *Euglena gracilis*, but could induce a 1.4-fold increase in the neutral lipids of all cells [25]. These studies indicate that inhibition of TOR activity may be one of the ways to increase TAG production in some algae. However, some studies have also shown that microalgal lipid biosynthesis varies greatly among different algal strains under environmental stress conditions [26].

Recently, different study groups have achieved a series of important results on the CsTOR signaling pathway through the use of TOR inhibitors (asTORis) and omics analysis in *Chlamydomonas reinhardtii* [17,27,28], but little is known about the TOR signaling pathway in *Chlorella sorokiniana* and the effects of TOR signaling on the growth and development of *Chlorella sorokiniana* has not been reported. In this study, we isolated and identified a strain of *Chlorella sorokiniana*, named it as *Chlorella sorokiniana* DP-1, and analyzed its nutritional composition. Drug sensitivity experiments showed that *Chlorella sorokiniana* DP-1 was insensitive to rapamycin, Torin1 and KU0063794, whereas AZD8055 could significantly inhibit the growth of *Chlorella sorokiniana* DP-1. Homologous sequence alignment and analysis showed that the conserved TOR signaling pathway components existed in the genome of *Chlorella sorokiniana*, including CsTOR, RAPTOR and LST8, indicating that the TOR signaling pathway is evolutionarily conserved in eukaryotes. RNA-seq analysis showed that CsTOR inhibition could alter the expression of genes related to photosynthesis and lipid and protein metabolism in *Chlorella sorokiniana* DP-1. Most genes involved in photosynthesis and carbon fixation were significantly downregulated, and almost all chlorophyll a-b binding protein, photosystem I and photosystem II genes were down-regulated in AZD8055-treated *Chlorella sorokiniana* DP-1, indicating that CsTOR positively regulated the photosynthesis of *Chlorella sorokiniana*. In addition, CsTOR controls protein synthesis and degradation by positively regulating ribosome synthesis and negatively regulating autophagy. However, most genes related to fatty acid degradation were upregulated, and most genes related to fatty acid synthesis were downregulated under CsTOR inhibition. Whether CsTOR negatively regulates lipid metabolism of *Chlorella sorokiniana* remains to be further investigated. Our study results suggest that CsTOR plays an important role in the photosynthesis and cellular metabolism of *Chlorella sorokiniana*.

## 2. Results

### 2.1. Isolation, Identification and Nutrition Analysis of Chlorella sorokiniana DP-1

We isolated and purified the collected samples and the species was identified. The isolated algal strain was preliminarily identified as Chlorella by morphology. ITS sequence analysis showed that the sequence coverage between the strain and *Chlorella sorokiniana* UTEX1665 was 98%, and the sequence similarity was 100%. An 18S rDNA sequence analysis showed that the sequence coverage between the strain and *Chlorella sorokiniana* (*Chlorella sorokiniana* strains KLL-G018, NIES 2173 and NKH18) was 100%, and the sequence similarity was 100%. Phylogenetic tree analysis of ITS and 18S rDNA fusion sequences showed that the strain belongs to *Chlorella sorokiniana* (Figure S1), and was named *Chlorella sorokiniana* DP-1.

**Figure 1:**
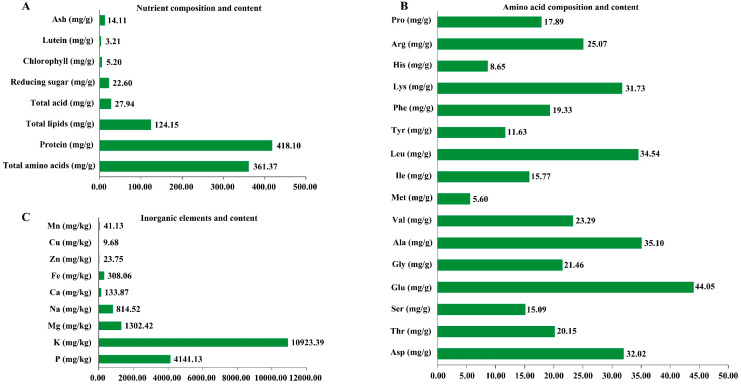
Nutritional composition and content of *Chlorella sorokiniana* DP-1. (**A**) Nutrient composition and content of *Chlorella sorokiniana* DP-1. (**B**) Amino acid composition and content of *Chlorella sorokiniana* DP-1. (**C**) Inorganic element and content of *Chlorella sorokiniana* DP-1. *Chlorella sorokiniana* DP-1 was cultured for 10 days, and the precipitated algae cells were collected by centrifugation. Nutritional compositions and contents were analyzed after drying.

We further analyzed the nutritional ingredients and contents of *Chlorella sorokiniana* DP-1. After culturing *Chlorella sorokiniana* DP-1 for 10 days, the precipitated algal cells were collected by centrifugation and made into dry algal powder, then its nutritional ingredients were analyzed. The results show that the total lipid content of *Chlorella sorokiniana* DP-1 was 12.42%, the protein content was 41.81% and the reducing sugar content was 2.26%. In addition, *Chlorella sorokiniana* DP-1 also contains a variety of mineral elements, vitamins and eight essential amino acids (Figure 1), indicating *Chlorella sorokiniana* DP-1 is a high-quality protein resource.

### 2.2. The Conserved TOR Signaling Pathway in Chlorella sorokiniana

**Figure 2:**
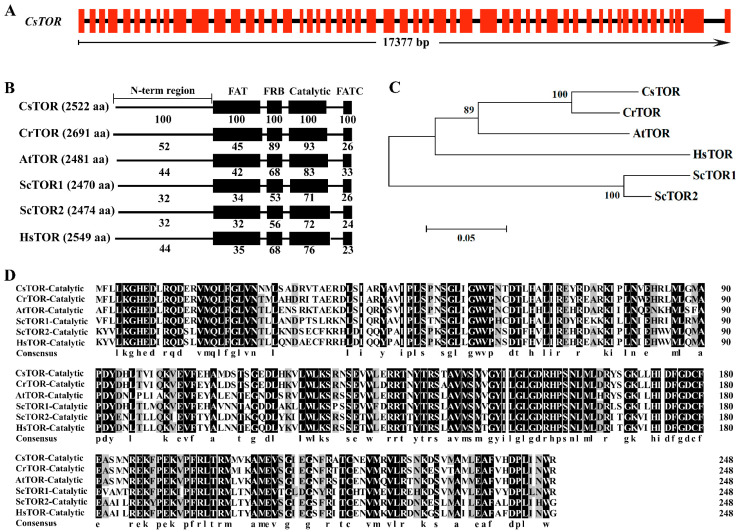
CsTOR structure and sequence analysis of *Chlorella sorokiniana*. (**A**) *CsTOR* gene sequence. The red rectangles represent exons. (**B**) The alignment of the CsTOR protein conserved domains with the TOR sequences of other species. The numerical value represents the similarity (%) of CsTOR protein sequence with that of other species, and the numbers in brackets represent the number of amino acids. *Chlorella sorokiniana* (Cs), *Chlamydomonas reinhardtii* (Cr), *Arabidopsis thaliana* (At), *Homo sapiens* (Hs), *Saccharomyces cerevisiae* (Sc). (**C**) Phylogenetic tree analysis of TOR protein sequences from *Chlorella sorokiniana* and other species. Phylogenetic tree generated from the neighbor-joining method (used MEGA 4 software, 1000 of bootstrap replicates) based on TOR protein sequences. (**D**) Sequence alignment analysis of the catalytic domain of CsTOR protein with that of other species.

As a master regulatory factor of growth and metabolism, the TOR signaling pathway is highly conserved in eukaryotes [29,30]. In order to identify whether there is an evolutionarily conserved TOR signaling pathway in *Chlorella sorokiniana*, BLASTp analysis was performed on the genome database of *Chlorella sorokiniana* UTEX 1230 with CrTOR signaling related proteins as the reference. CsTOR protein (Protein ID: 6516) was found in the genome of *Chlorella sorokiniana* (Table 1). The full length of the *CsTOR* gene was 17377 bp, including 52 introns and 53 exons, encoding 2522 amino acid residues, and has a molecular weight of 276 kDa (Figure 2A). Sequence alignment of TOR proteins between CsTOR and other species showed consistent conserved domains, including the N-terminal region, FAT, FRB, catalytic and FATC domains (Figure 2B). Phylogenetic analysis of CsTOR protein and alignment of the catalytic domain with other species (Figure 2C,D) showed that CsTOR was evolutionarily conserved, and CsTOR was most closely related to CrTOR evolutionarily. In addition, we also found other homologous genes encoding the TORC1 complex, including RAPTOR and LST8 (Table 1), but the key components of TORC2, RICTOR and SIN1, were absent in *Chlorella sorokiniana*. These results indicated that there may be only one functionally conserved TORC1 signaling in *Chlorella sorokiniana*.

**Figure 3:**
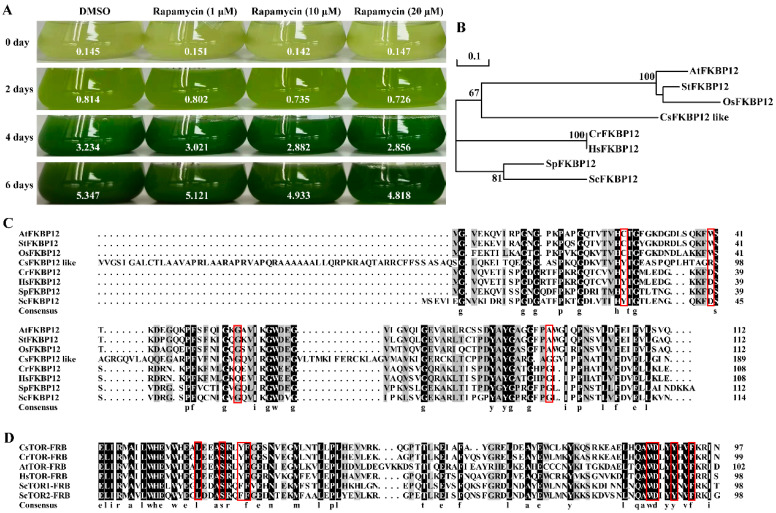
*Chlorella sorokiniana* cells are insensitive to rapamycin. (**A**) Phenotype of *Chlorella sorokiniana* DP-1 treated with different concentrations of rapamycin for 0, 2, 4 and 6 days. The values represent the corresponding OD680nm values. (**B**) Phylogenetic tree analysis of FKBP12 protein sequences from *Chlorella sorokiniana* and other species. Phylogenetic tree generated from the neighbor-joining method (used MEGA 4 software, 1000 of bootstrap replicates) based on TOR protein sequences. *Chlorella sorokiniana* (Cs), *Chlamydomonas reinhardtii* (Cr), *Arabidopsis thaliana* (At), *Homo sapiens* (Hs), *Saccharomyces cerevisiae* (Sc), *Schizosaccharomyces pombe* (Sp), *Oryza sativa* (Os), *Solanum tuberosum* (St). (**C**) Sequence alignment analysis of CsFKBP12 protein with that from other species. The red box represents the conserved amino acid of the FKBP12 binding to rapamycin. (**D**) Sequence alignment analysis of the FRB domain of CsTOR protein with that from other species. The red box represents the conserved amino acid of the FRB domain binding to rapamycin.

Rapamycin can combine with CrFKBP12 and CrTOR to form a ternary complex, resulting in significant inhibition of cell growth in *Chlamydomonas reinhardtii* [16]. However, the inhibition effect of rapamycin on *Chlorella sorokiniana* DP-1 was not obvious when treated with rapamycin at 1, 10 and 20 μM concentrations (Figure 3A and Figure S2). Sequence alignment analysis found that CsFKBP12 (Protein ID: 780), an ortholog of FKBP12, existed in *Chlorella sorokiniana*, which encodes a protein with 31% similarity to CrFKBP12 (Table 1). Phylogenetic analysis with other species indicated that CsFKBP12 was evolutionarily more closely related to FKBP12 of plants such as *Arabidopsis thaliana*, *Oryza sativa* and *Solanum tuberosum* (Figure 3B), all of which were insensitive to rapamycin due to the loss of function of the FKBP12 protein. Amino acid sequence alignment found that some conserved amino acid residues binding to rapamycin were mutated in the CsFKBP12 protein (Figure 3C), whereas the amino acids required for binding to rapamycin were highly conserved in the CsTOR-FRB domain (Figure 3D), indicating that the insensitivity of *Chlorella sorokiniana* DP-1 to rapamycin may be due to the loss of function of CsFKBP12 protein.

### 2.3. AZD8055 Inhibits the Growth of Chlorella sorokiniana in a Dose-Dependent Manner

**Figure 4:**
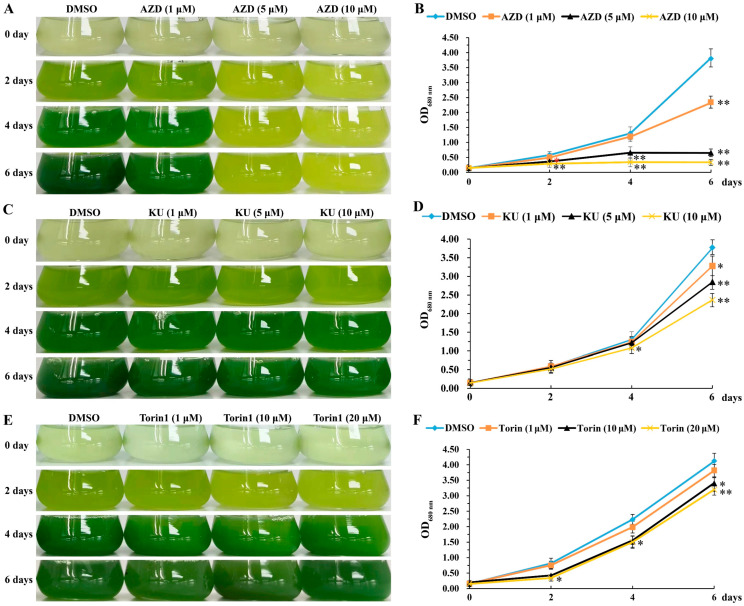
Effects of TOR kinase inhibitors on growth of *Chlorella sorokiniana* DP-1. (**A**) AZD inhibits the growth of *Chlorella sorokiniana* DP-1 in a dose-dependent manner. Phenotype of *Chlorella sorokiniana* DP-1 treated with different concentrations of AZD for 0, 2, 4 and 6 days. (**B**) Change curves of OD680nm values of *Chlorella sorokiniana* DP-1 treated with 1, 5 and 10 μM AZD for 0, 2, 4 and 6 days. (**C**) Phenotype of *Chlorella sorokiniana* DP-1 treated with different concentrations of KU for 0, 2, 4 and 6 days. (**D**) Change curves of OD680nm values of *Chlorella sorokiniana* DP-1 treated with 1, 5 and 10 μM KU for 0, 2, 4 and 6 days. (**E**) Phenotype of *Chlorella sorokiniana* DP-1 treated with different concentrations of Torin1 for 0, 2, 4 and 6 days. (**F**) Change curves of OD680nm values of *Chlorella sorokiniana* DP-1 treated with 1, 10 and 20 μM Torin1 for 0, 2, 4 and 6 days. The data represents the mean ± SD of *n* = 3 independent experiments. Significant differences between DMSO and treatments were indicated by one (* *p* < 0.05) or two (** *p* < 0.01) asterisks in two-tailed Student’s *t*-tests.

In order to explore the effect of novel inhibitors of TOR (asTORis) on the growth of *Chlorella sorokiniana*, *Chlorella sorokiniana* DP-1 was treated with AZD8055 (AZD) at concentrations of 1, 5 or 10 μM. The results found that AZD inhibited the growth of *Chlorella sorokiniana* in a dose-dependent manner, with a 50% inhibiting concentration (IC50) of about 1 μM and lethal concentration of about 5 μM AZD (Figure 4A,B). However, when *Chlorella sorokiniana* DP-1 was treated with KU0063794 (KU) at 1, 5 or 10 μM, or Torin1 at 1, 10 or 20 μM, there was no obvious difference in cell density between the treatment group and the control. Even high concentrations of KU or Torin1 only partially inhibited the growth of *Chlorella sorokiniana* DP-1 to a small extent (Figure 4C–F), indicating that the TOR inhibitors KU and Torin1 could hardly inhibit the growth of *Chlorella sorokiniana* DP-1. These results indicate that AZD8055, a specific inhibitor of TOR, can be used to analyze the function of CsTOR in *Chlorella sorokiniana*.

### 2.4. Analysis of Gene Expression Profile under CsTOR Inhibition

**Figure 5:**
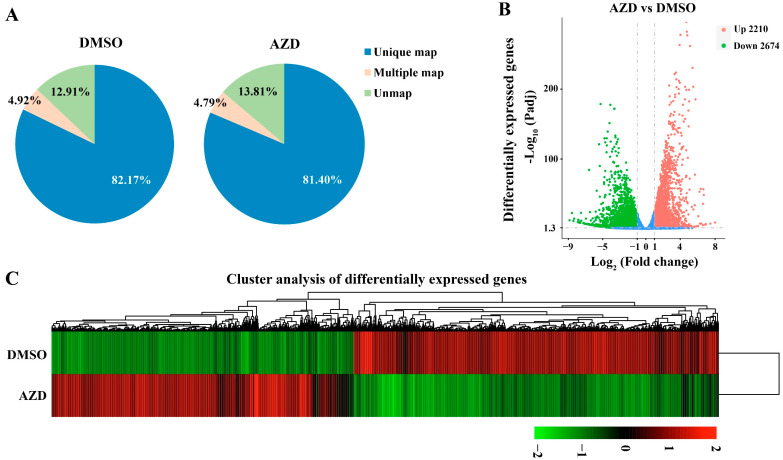
Transcriptome data analysis of *Chlorella sorokiniana* DP-1 under DMSO and AZD treatment. (**A**) Proportions of clean reads of unmapped, mapped to multiple genes and mapped to unique genes, which were plotted by three replicates of DMSO and AZD treatment. (**B**) The number of downregulated and upregulated differentially expressed genes for DMSO and AZD treatment. (**C**) Cluster analysis of differentially expressed genes for DMSO and AZD treatment. Red represents high gene abundance, and green represents low gene abundance.

In order to further elucidate the roles of CsTOR signaling in the growth of *Chlorella sorokiniana*, RNA-seq of *Chlorella sorokiniana* DP-1 was performed under CsTOR inhibition by AZD8055. Our previous experimental results found that 5 μM AZD8055 could completely inhibit the growth of *Chlorella sorokiniana* (Figure 4A,B), thus, RNA-seq was conducted in *Chlorella sorokiniana* DP-1 treated with 5 μM AZD8055 or an equal volume of DMSO as control. After filtering the raw data, clean reads were obtained for subsequent analysis. We found that over 85% of the clean reads could be mapped to the annotated genome of *Chlorella sorokiniana*, and more than 81% of the clean reads were uniquely mapped to the genome (Figure 5A). A total of 4884 differentially expressed genes (DEGs) were found between the AZD treatment group and the DMSO control group, of which 2210 DEGs were upregulated and 2674 DEGs were downregulated (Figure 5B). In addition, hierarchical cluster analysis of DEGs was performed. The results show that the expression levels of many genes in *Chlorella sorokiniana* were changed after AZD treatment compared with DMSO (Figure 5C). Further, some DEGs were randomly selected from the RNA-seq data, and the reliability of the RNA-seq data was verified by quantitative real-time PCR. RT-PCR results show the same trend as obtained in RNA-seq data (Figure S3), indicating that the RNA-seq data were reliable and valid.

### 2.5. Gene Ontology (GO) and KEGG Pathway Enrichment Analysis of DEGs

**Figure 6:**
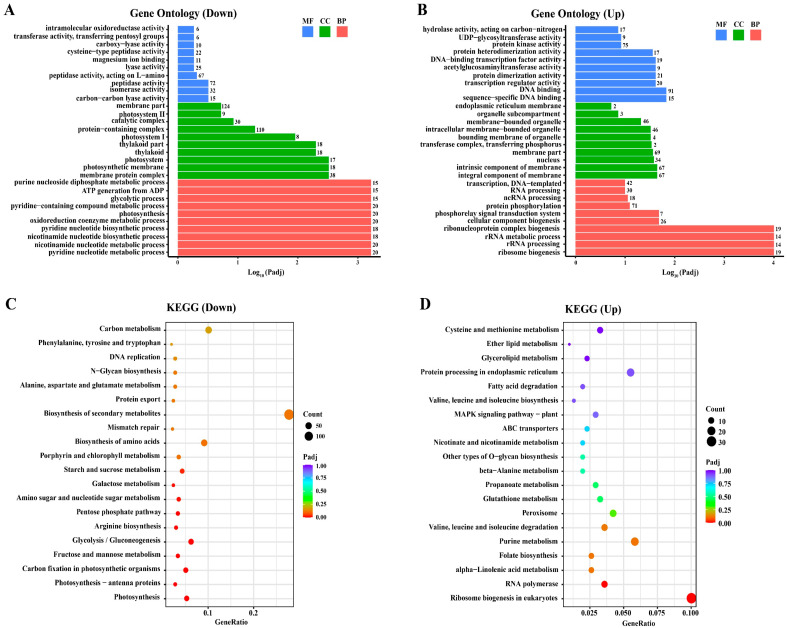
Gene ontology and KEGG pathway enrichment analysis of DEGs. (**A**) Significantly downregulated enriched gene ontology for AZD treatment in the RNA-seq database. (**B**) Significantly upregulated enriched gene ontology for AZD treatment in the RNA-seq database. Gene ontology was ranked by their significance. The value represents the number of differentially expressed genes. MF, molecular function; CC, cellular component; BP, biological process. (**C**) The top 20 functionally enriched KEGG pathways in downregulated DEGs. (**D**) The top 20 functionally enriched KEGG pathways in upregulated DEGs.

In order to analyze the functions of DEGs and to understand the effect of CsTOR on the growth and development in *Chlorella sorokiniana*, gene ontology (GO) functional enrichment and KEGG pathway enrichment analysis of DEGs were performed. GO is a comprehensive database describing gene functions, including biological process, cellular component and molecular function. A total of 594 downregulated GO terms and 520 upregulated GO terms were enriched in the RNA-seq data. Among the downregulated GO terms, the most significantly enriched GO terms in biological process were pyridine nucleotide metabolic process and photosynthesis, the most significantly enriched GO terms in cellular component were photosynthetic membrane and photosystem, and the most significantly enriched GO terms in molecular function were carbon-carbon lyase activity and peptidase activity (Figure 6A). Among the upregulated GO terms, the most significantly enriched GO terms in biological process were ribosome biogenesis and rRNA processing, in cellular component were integral component of membrane and intrinsic component of membrane, and in molecular function were sequence-specific DNA binding and DNA binding (Figure 6B). These results indicate that the inhibition of CsTOR by AZD changed the expression of genes related to photosynthesis and other biological processes.

To analyze the specific metabolic processes and signal transduction pathways in which DEGs were involved, the KEGG (Kyoto Encyclopedia of Genes and Genomes) database was used to analyze the DEGs. Among downregulated DEGs, the most significantly enriched three KEGG pathways were photosynthesis, photosynthesis-antenna proteins and carbon fixation in photosynthetic organisms (Figure 6C). Among upregulated DEGs, the most significantly enriched three KEGG pathways were ribosome biogenesis in eukaryotes, RNA polymerase and alpha-linolenic acid metabolism (Figure 6D). These results indicate that CsTOR regulates multiple metabolic processes and signal transduction pathways in *Chlorella sorokiniana*.

### 2.6. CsTOR Regulates Chloroplast Development and Photosynthesis in Chlorella sorokiniana

Green plants can carry out photosynthesis because they all contain chloroplasts, an organelle that can complete energy conversion. Chloroplast biogenesis and development plays a key role in photosynthetic efficiency [31,32]. Previous studies have shown that the chlorophyll catabolism genes *STAY-GREEN 1* (*SGR1*) and *NON-YELLOW COLORING 1* (*NYC1*) can promote chlorophyll catabolism in chloroplasts [33,34]. Transcriptomic data shows that 25 DEGs were assigned to the “Porphyrin and chlorophyll metabolism” KEGG pathway under CsTOR inhibition. It is worth noting that the chlorophyll catabolism genes *CsSGR1* (*gene_3161*) and *CsNYC1* (*gene_11101*) were upregulated 10.56- and 24.08-fold, respectively, whereas the porphyrin and chlorophyll synthesis genes were significantly downregulated (Supplementary Table S1). In addition, thylakoid is a key structure of chloroplast photosynthesis, which contains photosynthetic pigments and electron transport chain components. We found that all 18 DEGs on the thylakoid were downregulated under CsTOR inhibition (Supplementary Table S1). These results indicate that CsTOR positively regulates chloroplast development.

**Figure 7:**
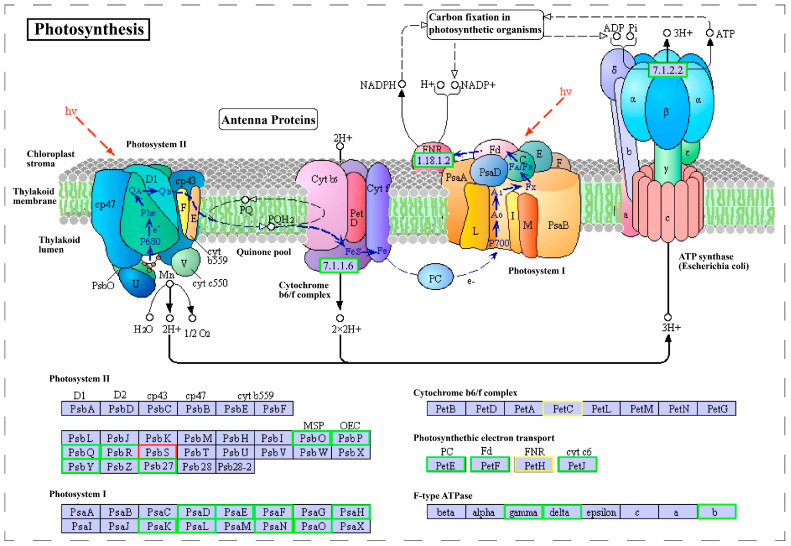
DEGs involved in the regulation of photosynthesis in *Chlorella sorokiniana* DP-1. Green boxes represent downregulated genes, red boxes indicate upregulated genes, and yellow boxes represent both up- and down-regulated genes.

Photochemical reactions in photosynthesis are performed by two photosystems including photosystem I (PSI) and photosystem II (PSII). Studies have shown that inhibition of CrTOR activity results in a low photosynthetic electron transport rate in *Chlamydomonas reinhardtii* cells and reduces the photosynthetic efficiency of PSI and PSII in chloroplasts [35]. In this study, RNA-seq data analysis shows that the change of photosynthesis was the most significant in GO terms and KEGG pathways (Figure 6). A total of 74 DEGs related to photosynthesis were found in the RNA-seq data, of which 30 DEGs were enriched in the KEGG “Photosynthesis” pathway, 14 DEGs were enriched in the KEGG “Photosynthesis—antenna proteins” pathway, and 30 DEGs were enriched in the KEGG “Carbon fixation in photosynthetic organisms” pathway (Table 2). Moreover, most genes involved in photosynthesis and carbon fixation, such as chlorophyll a-b binding protein, were significantly downregulated in *Chlorella sorokiniana* treated with AZD (Table 2). Importantly, almost all genes of chlorophyll a-b binding protein, photosystem I and photosystem II were downregulated (Figure 7), indicating that CsTOR inhibition significantly downregulated the expression of photosynthesis-related genes in *Chlorella sorokiniana*. These results indicate that CsTOR positively regulates chloroplast development and photosynthesis in *Chlorella sorokiniana*.

### 2.7. CsTOR Regulates Lipid and Protein Metabolism in Chlorella sorokiniana

Previous studies found that the formation of lipid droplets and accumulation of triacylglycerols can be induced by the TOR inhibitor Torin1 or AZD8055 in *Chlamydomonas reinhardtii* cells [23]. Analysis of DEGs revealed that the TOR signaling pathway regulates lipid metabolism in *Chlorella sorokiniana* DP-1. A total of 17 DEGs were assigned to lipid metabolism, of which 9 DEGs were enriched in the KEGG “fatty acid degradation” pathway and 8 DEGs were enriched in the KEGG “fatty acid biosynthesis” pathway. Importantly, most genes related to fatty acid degradation were upregulated, whereas most genes related to fatty acid synthesis were significantly downregulated in *Chlorella sorokiniana* DP-1 cells treated with AZD (Supplementary Table S2), implying that CsTOR positively regulated the biosynthesis of fatty acids.

Ribosomes composed of rRNA and ribosomal proteins are responsible for protein synthesis in all cells [36,37]. In this study, we found that 33 DEGs involved in the regulation of ribosomal proteins were enriched in the “ribosome” KEGG pathway, including 25 downregulated DEGs and 8 upregulated DEGs (Supplementary Table S2). The most downregulated gene was *40S ribosomal protein S26* (*gene_8933*) at 63.12-fold. These results suggested that the inhibition of CsTOR leads to the dysfunction of ribosomes, which in turn affects protein synthesis. Autophagy is an important pathway for protein degradation and numerous studies have shown that TOR negatively regulates autophagy [38,39,40]. In this study, autophagy-related genes were significantly upregulated under CsTOR inhibition, such as the *CsATG5* (*gene_11198*) and *CsATG13* (*gene_11328*) genes that were upregulated 5.03- and 2.08-fold, respectively. However, the *CsPP2A* (*gene_3520*), a negative regulatory gene of autophagy, was significantly downregulated by 5.06-fold. These results indicate that CsTOR regulates protein synthesis and degradation by controlling ribosome synthesis and autophagy.

## 3. Discussion

TOR is a conserved protein kinase in all eukaryotes that plays a central regulating role in cell proliferation, growth and metabolism [9,11,12,29]. At present, many achievements have been made in the TOR signaling pathway of the model algae *Chlamydomonas reinhardtii*, but whether there is a conserved TOR signaling pathway in *Chlorella sorokiniana* has not been reported. Analysis of the CsTOR signaling pathway is important for gaining insight into the cell growth of *Chlorella sorokiniana*. In this study, we isolated and identified a strain of *Chlorella sorokiniana*, and named it as *Chlorella sorokiniana* DP-1. The total lipid content was 12.42%, protein content was 41.81% and reducing sugar content was 2.26% in *Chlorella sorokiniana* DP-1 (Figure 1). Based on sequence alignment analysis, it was found that there was a conserved TOR protein in the *Chlorella sorokiniana* genome database (Figure 2). In addition, we also found other homologous genes encoding TORC1, including RAPTOR and LST8 (Table 1). Consistent with other studies in plants, TORC2 was also absent in *Chlorella sorokiniana*. These results indicated that there may be only one functionally conserved TORC1 signaling in *Chlorella sorokiniana*.

TOR is a key regulatory center for cell growth and development, and specific inhibitors of TOR proteins have been developed, including rapamycin and inhibitors targeting the TOR kinase domain (asTORis). In the model algae *Chlamydomonas reinhardtii*, 100 nM of rapamycin can inhibit cell growth. The inhibitory effect of rapamycin on the growth of *Chlamydomonas reinhardtii* cells increased with increasing concentration [16]. In this study, we found that the sensitivity of *Chlorella sorokiniana* to rapamycin was inconsistent with that of *Chlamydomonas reinhardtii*. Rapamycin, up to 20 μM, had no obvious inhibiting effect on the growth of *Chlorella sorokiniana* DP-1 (Figure 3). Compared with rapamycin, asTORis have a higher inhibiting efficiency on TOR in plants. Torin1, KU0063794, PP242 and AZD8055 have been widely used in the studies on TOR function in plants, and the application of asTORis directly promoted the analysis of plant TOR function [20,21,22]. The IC50 value of AZD8055 inhibiting TOR kinase activity was 0.8 nM in mammalian cells [23] and the IC50 value was 1 μM AZD8055 in *Arabidopsis thaliana* [41]. In this study, we found that AZD8055 inhibited the growth of *Chlorella sorokiniana* in a dose-dependent manner, with an IC50 concentration of about 1 μM and a lethal concentration of about 5 μM (Figure 4A,B). Unexpectedly, *Chlorella sorokiniana* DP-1 was almost insensitive to Torin1 and KU0063794 (Figure 4C–F), and the underlying reasons still need to be further studied. In order to further clarify the role of the CsTOR signaling pathway in the growth of *Chlorella sorokiniana*, we performed transcriptome sequencing under CsTOR inhibition with AZD8055. GO enrichment and KEGG pathway analysis of DEGs showed that CsTOR regulated various biological metabolic processes and signal transduction pathways, especially CsTOR plays an important role in photosynthesis and lipid and protein metabolism (Figure 6).

*Chlorella sorokiniana* is widely distributed in freshwater environments and has the advantages of fast growth, high lipid content and strong adaptability to different extreme environments. *Chlorella sorokiniana* can be used for large-scale culture and production of biomass, oil and other bulk raw materials, which have important industrial value in biofuel production [2,42,43]. However, microalgae produced only small amount of lipids under favorable environmental conditions [44]. Environmental stress often inhibits photosynthesis and cell growth, which promotes the accumulation of lipids in microalgae. Studies have shown that lipid biosynthesis in microalgae induced by environmental stress varies greatly between different algal strains and species [26]. For example, TOR inhibitor Torin1 or AZD8055 induced the formation of lipid droplets and the accumulation of triacylglycerols in *Chlamydomonas reinhardtii* cells [19]. However, in this study, we found that most of the genes related to fatty acid degradation were upregulated, whereas most of the genes related to fatty acid synthesis were downregulated under CsTOR inhibition, indicating that AZD treatment may inhibit lipid biosynthesis in *Chlorella sorokiniana* DP-1, but the underlying reason remains to be investigated. Interestingly, TOR positively regulates fatty acid biosynthesis in mammals, implying that CsTOR and mammalian TOR have similar functions in fatty acid metabolism.

## 4. Materials and Methods

### 4.1. Isolation, Culture and Identification of Chlorella sorokiniana DP-1

Samples were collected from soil in a greenhouse base in Chengdu, China. In the laboratory, the soil samples were first placed in a 50 mL centrifuge tube, and 30 mL of BG11 culture solution was added to dissolve samples fully. After screening out large microorganisms and impurities with gauze, the samples were cultured in a constant temperature shaker at 28 °C, 150 rpm and 100 μmol m^−2^ s^−1^ light density for several days. The algae in the sample were separated and purified by the dilution separation method. A total of 200 μL of the diluted sample was taken and evenly spread onto BG-11 solid medium including 250 mg/L cephalosporin, and then placed in a constant temperature light incubator for a period of time (temperature 28 °C, 100 μmol m^−2^ s^−1^ light density). The growth of single algal colonies was carefully observed during the culture process. The sterile single algal colonies on the solid medium were selected and placed into centrifuge tubes containing 800 μL of BG-11 liquid medium, and these centrifuge tubes were placed in the above-mentioned constant temperature light incubator for continued culture under the same conditions. Species identification was performed for the pure algal species obtained after separation and purification, which mainly relied on microscopic observation and molecular biological means [45,46,47]. The 18S rDNA and ITS sequences of *Chlorella sorokiniana* DP-1 were amplified by PCR with specific primers. The 18S rDNA and ITS sequences of *Chlorella sorokiniana* DP-1 were analyzed in the NCBI database, and the phylogenetic tree was constructed by the neighbor-jointing method from MEGA7.1 software. The primers used to amplify 18S rDNA and ITS sequences are shown in Supplementary Table S3.

### 4.2. Nutritional Composition Analysis of Chlorella sorokiniana DP-1

*Chlorella sorokiniana* DP-1 was cultured in BG11 liquid medium containing 10 g/L glucose. *Chlorella sorokiniana* DP-1 was cultured in a constant temperature shaker at 28 °C, 150 rpm and 100 μmol m^−2^ s^−1^ light density for 10 days. The precipitated algal cells were collected by centrifugation and made into a dry algal powder; then, its nutritional ingredients were analyzed, including Ca, K, Na, Mg, Fe, Mn, P, Zn, Cu, ash, protein, reducing sugar, total acid, amino acid components, vitamins, chlorophyll, fatty acid and lutein. Mineral elements in *Chlorella sorokiniana* DP-1 were determined by inductively coupled plasma optical emission spectrometer (iCAP™ PRO ICP-OES, Thermo Scientific, Waltham, MA, USA), and the test method was referred to GB5009268-2016. Amino acid components were determined using an automatic amino acid analyzer (MembraPure A300, membraPure GmbH, Hennigsdorf, Germany), and the test method was referred to GB 5009124-2016. The determination of total lipid content was performed by gas chromatograph–mass spectrometer (Agilent 7890B+5977A, Agilent Technologies Inc., Santa Clara, CA, USA). The chlorophyll content was determined by spectrophotometry according to NY/T3082-2017, the protein content was determined by the Kjeldahl method according to GB50095-2016, and the reducing sugar content was determined by direct titration according to GB50097-2016. The determination of ash and total acid contents refer to GB50094-2016 and GB12456-2021, respectively. Each index was repeated three times, and the specific experimental operation was carried out by the China National Institute of Testing technology.

### 4.3. Effects of TOR Inhibitors on the Growth of Chlorella sorokiniana DP-1

*Chlorella sorokiniana* DP-1 was cultured in BG11 liquid medium containing 10 g/L glucose. *Chlorella sorokiniana* DP-1 was cultured in a constant temperature shaker at 28 °C, 150 rpm and 100 μmol m^−2^ s^−1^ light density for 7 days. The algal solution was then inoculated into a new 50 mL BG11 medium at 1% volume. AZD8055 or KU was added to a final concentration of 1, 5 or 10 μM, and the final concentrations of Torin1 and RAP were 1, 10 and 20 μM. The OD680nm values of *Chlorella sorokiniana* DP-1 were measured by a spectrophotometer (Biotek EpochTM2, BioTECH, Winooski, VT, USA) at 0, 2, 4 and 6 days. A total of 200 µL treated *Chlorella sorokiniana* DP-1 was added into a 96-well plate, then put it into the spectrophotometer to detect the absorbance of D680nm at 28 °C. Three biological replicates were performed for each treatment.

### 4.4. Total RNA Extraction and Detection for Transcriptome Sequencing

*Chlorella sorokiniana* DP-1 was cultured in BG11 liquid medium containing 10 g/L glucose. *Chlorella sorokiniana* DP-1 was cultured in a constant temperature shaker at 28 °C, 150 rpm and 100 μmol m^−2^ s^−1^ light density for 7 days, and then treated with 5 μM AZD8055 or an equal volume DMSO (as a control) for 24 h. For each treatment, three independent biological replicates were performed. The precipitated algal cells were collected by centrifugation. Then, the total RNAs of *Chlorella sorokiniana* DP-1 treated with AZD and DMSO were extracted using the Hipure Plant RNA Kit (Magen, Guangzhou, China). The concentration and integrity of extracted RNA were detected with a Nano Photometer spectrophotometer and Agilent 2100 bioanalyzer.

### 4.5. RNA Library Construction and Transcriptome Sequencing

The qualified total RNA was used for the construction of transcriptome sequencing library, and the total content was over 1 μg. The cDNA library was constructed using Illumina’s NEBNext^®^ UltraTMRNA Library Prep Kit. After passing the quality control, the library preparations were sequenced on an Illumina Novaseq platform and 150 bp paired-end reads were generated.

### 4.6. Reads Mapping to the Reference Genome

Clean reads were obtained after removing the containing adapter and low-quality reads from raw data. At the same time, Q20, Q30 and GC content in the clean data were calculated. Download the *Chlorella sorokiniana* UTEX 1230 reference genome and gene annotation files from the NCBI website (https://ftp.ncbi.nlm.nih.gov/genomes/all/GCA/003/130/725/GCA_003130725.1_ASM313072v1/; accessed on 4 June 2022). The clean reads were mapped to the reference genome using the HISAT2 software.

### 4.7. Differentially Expressed Genes, GO and KEGG Enrichment Analysis

Quantitative analysis of genes was performed for each sample with the Feature Counts software, and the FPKM value of each gene was calculated. Differentially expressed genes between AZD and DMSO treatment (3 biological replicates per treatment) were screened with DESeq2 R software [48]. Corrected P-adj < 0.05 and |Log2 (Fold change)| > 1 were set as the thresholds for differentially expressed genes.

GO (Gene Ontology) and KEGG (Kyoto Encyclopedia of Genes and Genomes) enrichment analysis of differentially expressed genes were realized using Cluster Profiler R software. The corrected *p* value < 0.05 was significant enrichment of GO or KEGG.

### 4.8. Quantitative Real-Time PCR

To test the reliability of transcriptome data, 15 differentially expressed genes were randomly selected for quantitative real-time PCR verification. Total RNA extraction, reverse transcription and quantitative PCR were performed on *Chlorella sorokiniana* DP-1 treated with the same batch as that used for transcriptome sequencing. *CsACTIN* (*gene_5878*) was used as an internal reference gene. RNA relative quantification analyses were performed with the formula: 2^−∆∆CT^. The data were expressed as the mean ± SD of three independent experiments. Primers for quantitative real-time PCR were shown in Supplementary Table S3.

### 4.9. Statistical Analysis

Two-tailed Student’s *t*-tests were performed using the IBM SPSS statistics software to investigate the significant differences between control and treatments. Significant differences between control and treatments were indicated by one (* *p* < 0.05) or two (** *p* < 0.01) asterisks in the two-tailed Student’s *t*-tests.

## 5. Conclusions

**Figure 8:**
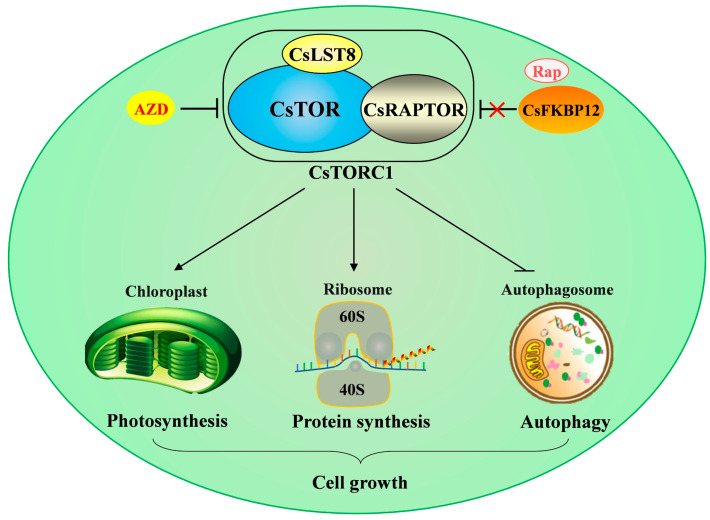
The graphic illustration of CsTOR signaling regulating cell growth in *Chlorella sorokiniana*. Arrows and T-bars represent enhancement and inhibition, respectively. AZD, AZD8055; Rap, rapamycin.

In conclusion, this study elucidated the conserved TOR signaling pathway in *Chlorella sorokiniana* and clarified the effect of TOR inhibitors on the growth of *Chlorella sorokiniana*. Consistent with other plants, *Chlorella sorokiniana* was insensitive to rapamycin due to the lack of functional FKBP12. Interestingly, *Chlorella sorokiniana* was also insensitive to Torin1 and KU0063794, whereas AZD8055 could significantly inhibit the growth of *Chlorella sorokiniana*. Transcriptome sequencing analysis indicated that CsTOR played important roles in chloroplast formation, photosynthesis and lipid and protein metabolism (Figure 8). These observations provide new insights into the function of CsTOR in *Chlorella sorokiniana*.

## Figures and Tables

**Table 1:** The putative components of TOR signaling pathway in *Chlorella sorokiniana*.

Protein Name	*Chlamydomonas reinhardtii*	*Chlorella sorokiniana*	Identity (%)
Target of rapamycin (TOR)	CrTOR	CsTOR (Protein ID: 6516)	57
Regulatory associate protein of TOR (RAPTOR)	CrRAPTOR	CsRAPTOR (Protein ID: 11673)	35
Lethal with SEC-13 protein 8 (LST8)	CrLST8	CsLST8 (Protein ID: 10108)	79
FK506-binding protein 12 (FKBP12)	CrFKBP12	CsFKBP12 (Protein ID: 780)	31
Ribosomal protein S6 kinase (S6K)	CrS6K	CsS6K (Protein ID: 467)	40
Ribosome protein small subunit 6 (RPS6)	CrRPS6	CsRPS6 (Protein ID: 9527)	80
Transcription factor E2F alpha (E2FA)	CrE2FA	CsE2FA (Protein ID: 7062)	37
Translation initiation factor 2 alpha subunit (eIF2α)	CreIF2α	CseIF2α (Protein ID: 5064)	63
Type-2A-phosphatase-associated protein 46 (TAP46)	CrTAP46	CsTAP46 (Protein ID: 1579)	38
Brassinosteroid-insensitive 2 (BIN2)	CrBIN2	CsBIN2 (Protein ID: 10292)	59
Autophagy protein 1 (ATG1)	CrATG1	CsATG1 (Protein ID: 3352)	37
Autophagy protein 13 (ATG13)	CrATG13	CsATG13 (Protein ID: 11328)	32

**Table 2:** Differentially expressed genes in photosynthetic process in the RNA-seq data.

Gene ID	Log_2_ (Fold Change)	P-adj	Description
**Photosynthesis**			
*Gene_7859*	−4.2295	9.77 × 10^−152^	Photosystem I reaction center subunit III
*Gene_6838*	−4.5498	3.77 × 10^−130^	Oxygen evolving enhancer protein 3
*Gene_1173*	−4.4406	1.03 × 10^−139^	Photosystem I reaction center subunit XI
*Gene_7018*	−4.1567	1.22 × 10^−103^	Plastocyanin
*Gene_989*	−4.0112	1.14 × 10^−11^	Photosystem I reaction center subunit N (PSI-N)
*Gene_12768*	−3.8522	1.31 × 10^−22^	Oxygen-evolving enhancer protein 1, chloroplastic
*Gene_10589*	−3.7829	4.68 × 10^−63^	Oxygen-evolving enhancer protein 2, chloroplastic
*Gene_7696*	−3.7137	1.34 × 10^−2^	Photosystem I reaction center subunit VI
*Gene_10201*	−3.6719	8.39 × 10^−118^	Photosystem I reaction center subunit IV/PsaE
*Gene_2740*	−3.6205	1.50 × 10^−8^	Ferredoxin I
*Gene_5865*	−3.4432	6.71 × 10^−122^	Photosystem I reaction center subunit psaK
*Gene_9453*	−3.3892	4.31 × 10^−17^	PsbP-like protein 1, chloroplastic
*Gene_4661*	−3.1879	9.23 × 10^−73^	Photosystem II 10 kDa polypeptide PsbR
*Gene_4210*	−2.8369	7.67 × 10^−82^	Photosystem II core complex proteins psbY
*Gene_102*	−2.8317	4.70 × 10^−41^	Photosystem I subunit O
*Gene_8562*	−2.7363	1.41 × 10^−27^	Cytochrome C oxidase, cbb3-type, subunit III
*Gene_2471*	−2.5002	9.71 × 10^−58^	Ferredoxin--NADP reductase
*Gene_4149*	−2.4831	8.98 × 10^−74^	Photosystem I reaction center subunit II
*Gene_1323*	−2.2436	3.37 × 10^−33^	Photosystem II Pbs27
*Gene_5304*	−1.9695	4.70 × 10^−17^	Ferredoxin
*Gene_4612*	−1.6795	1.01 × 10^−46^	ATP synthase gamma chain, chloroplastic
*Gene_5593*	−1.5767	1.38 × 10^−24^	Ferredoxin
*Gene_770*	−1.4817	3.18 × 10^−14^	2Fe-2S iron-sulfur cluster binding domain
*Gene_1305*	−1.2470	3.86 × 10^−24^	ATP synthase subunit b
*Gene_2652*	−1.2397	1.29 × 10^−21^	Cytochrome b6-f complex iron-sulfur subunit 1
*Gene_2721*	−1.1797	9.32 × 10^−26^	ATP synthase delta chain, chloroplastic
*Gene_11903*	−1.0811	4.66 × 10^−3^	Cytochrome c6, chloroplastic
*Gene_3214*	1.7080	2.78 × 10^−26^	Cytochrome b6-f complex iron-sulfur subunit
*Gene_11015*	3.6736	4.25 × 10^−38^	Ferredoxin--NADP reductase, chloroplastic
*Gene_7461*	6.6924	3.58 × 10^−57^	Photosystem II 22 kDa protein, chloroplastic
**Photosynthesis—antenna proteins**			
*Gene_704*	−5.2606	3.95 × 10^−179^	Photosystem I chlorophyll a/b-binding protein 3-1
*Gene_4943*	−5.7198	4.16 × 10^−55^	Chlorophyll a-b binding protein CP26
*Gene_10267*	−4.9940	8.92 × 10^−38^	Chlorophyll a-b binding protein CP29
*Gene_7745*	−4.5441	7.80 × 10^−120^	Photosystem I chlorophyll a/b-binding protein 5
*Gene_10399*	−4.3288	5.87 × 10^−32^	Chlorophyll a-b binding protein 151, chloroplastic
*Gene_410*	−4.1943	1.76 × 10^−20^	Photosystem I chlorophyll a/b-binding protein 5
*Gene_10700*	−4.0892	2.44 × 10^−125^	Chlorophyll a-b binding protein 4, chloroplastic
*Gene_1778*	−3.7550	1.37 × 10^−95^	Chlorophyll a-b binding protein P4, chloroplastic
*Gene_6489*	−3.3811	5.45 × 10^−21^	Chlorophyll a-b binding protein of LHCII type I
*Gene_4912*	−3.3601	5.29 × 10^−105^	Chlorophyll a-b binding protein 1B-21
*Gene_6490*	−3.3371	4.80 × 10^−58^	Chlorophyll a-b binding protein of LHCII type I
*Gene_7746*	−3.3230	1.01 × 10^−57^	Chlorophyll a-b binding protein P4, chloroplastic
*Gene_4913*	−3.1729	1.53 × 10^−82^	Chlorophyll a-b binding protein P4, chloroplasti
*Gene_8524*	−2.7988	3.58 × 10^−2^	Chlorophyll a-b binding protein type 2 member 1B
**Carbon fixation in photosynthetic organisms**			
*Gene_5465*	−3.1879	2.35 × 10^−73^	Ribulose bisphosphate carboxylase small chain 5
*Gene_10970*	−3.3100	4.95 × 10^−59^	Phosphoglycerate kinase, chloroplastic
*Gene_4119*	−3.1352	2.16 × 10^−14^	Fructose-bisphosphate aldolase 6
*Gene_11119*	−2.8156	1.98 × 10^−43^	Glyceraldehyde-3-phosphate dehydrogenase 2
*Gene_8254*	−2.7483	8.03 × 10^−20^	Ribose 5-phosphate isomerase A
*Gene_11481*	−2.4254	4.76 × 10^−61^	Phosphoglycerate kinase
*Gene_7269*	−2.3419	7.11 × 10^−44^	Triosephosphate isomerase
*Gene_7591*	−2.3249	1.43 × 10^−39^	Fructose-1-6-bisphosphatase
*Gene_3067*	−2.2774	4.06 × 10^−70^	Phosphoenolpyruvate carboxykinase
*Gene_9410*	−2.2702	1.17 × 10^−60^	Fructose-1-6-bisphosphatase
*Gene_9161*	−2.0867	5.88 × 10^−54^	Phosphoribulokinase, chloroplastic
*Gene_3113*	−1.9230	4.01 × 10^−31^	Alanine aminotransferase 2
*Gene_766*	−1.9038	5.59 × 10^−26^	Ribose 5-phosphate isomerase A
*Gene_7161*	−1.8384	5.10 × 10^−53^	Phosphoenolpyruvate carboxylase 2
*Gene_9959*	−1.8202	7.78 × 10^−33^	Glyceraldehyde-3-phosphate dehydrogenase A
*Gene_10788*	−1.7677	4.57 × 10^−38^	Fructose-bisphosphate aldolase 6
*Gene_3132*	−1.7541	7.38 × 10^−26^	Malate dehydrogenase
*Gene_3382*	−1.7454	2.82 × 10^−35^	Aspartate aminotransferase P2
*Gene_10495*	−1.7281	3.03 × 10^−18^	Phosphoenolpyruvate carboxylase 1
*Gene_142*	−1.6720	3.74 × 10^−28^	NAD-dependent malic enzyme 2
*Gene_7162*	−1.6695	4.47 × 10^−22^	Phosphoenolpyruvate carboxylase 2
*Gene_12754*	−1.4082	7.69 × 10^−32^	Fructose-bisphosphate aldolase 1
*Gene_7707*	−1.2021	1.86 × 10^−2^	Aminotransferase class I and II
*Gene_318*	−1.1383	8.17 × 10^−8^	Fructose-1,6-bisphosphatase
*Gene_10729*	−1.0475	1.22 × 10^−15^	Malate dehydrogenase
*Gene_2262*	−1.0192	4.81 × 10^−2^	Fructose-bisphosphate aldolase 8
*Gene_1724*	1.0143	7.55 × 10^−9^	Las17-binding protein actin regulator
*Gene_6684*	1.3748	2.87 × 10^−30^	Lactate/malate dehydrogenase
*Gene_9049*	1.6966	1.13 × 10^−53^	NADP-dependent malic enzyme
*Gene_1265*	1.6985	2.14 × 10^−11^	Phosphoenolpyruvate carboxykinase

## Data Availability

The ITS and 18S rDNA sequences identified by Chlorella sorokiniana DP-1 have been submitted to NCBI GenBank with accession numbers ON568499 and ON566028, respectively. The RNA-seq data have been deposited in the NCBI Sequence Read Archive under accession number PRJNA841783.

## Supplementary Materials

The following supporting information can be downloaded at: https://www.mdpi.com/article/10.3390/ijms23137451/s1.
