# Supplementary material for: Target of Rapamycin Signaling Involved in the Regulation of Photosynthesis and Cellular Metabolism in Chlorella sorokiniana
Source: Int J Mol Sci. 2022 Jul 4;23(13):7451. doi: 10.3390/ijms23137451 (PMC9266951; doi:10.3390/ijms23137451)
Supplement: Supplementary file 1 [file ijms-23-07451-s001.zip › ijms-1780034-supplementary.pdf]

International Journal of *Molecular Sciences*

MDPI

Article

Target of Rapamycin Signaling Involved in the Regulation of Photosynthesis and Cellular Metabolism in *Chlorella sorokiniana*

Linxuan Li †, Tingting Zhu †, Lele Huang and Maozhi Ren *

Supplementary materials

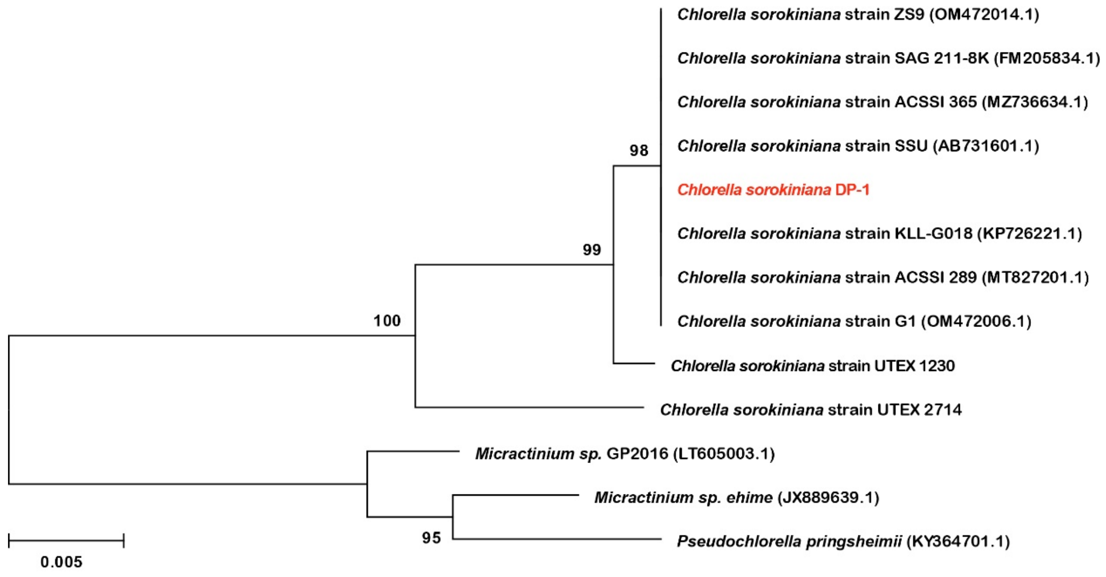

Figure S1. Phylogenetic tree generated from the Neighbor-Joining method (used MEGAX, 1000 of bootstrap replicates) based on the ITS and 18S rDNA fusion sequences. Parsimony bootstrap values of more than 50% are shown at the nodes. The isolate *Chlorella sorokiniana* DP-1 strain in this study was clustered the *Chlorella sorokiniana*.

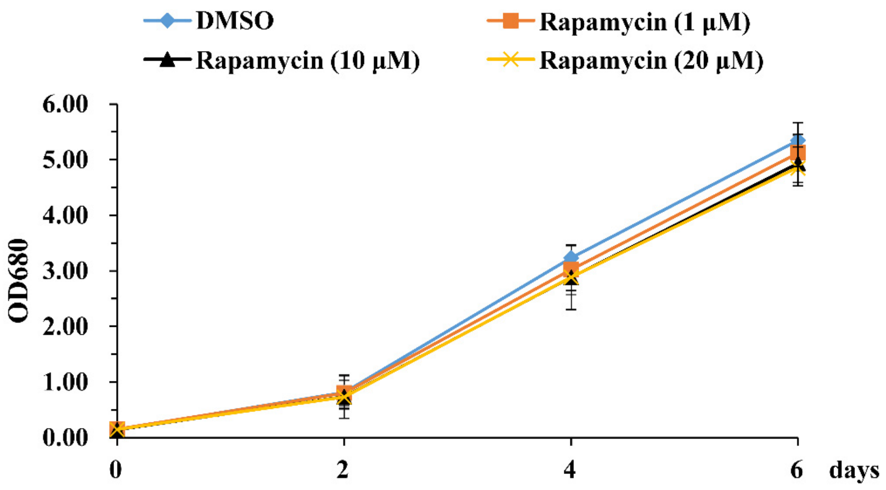

Figure S2. Change curves of OD680nm values of *Chlorella sorokiniana* DP-1 treated with 1, 10 and 20 μM rapamycin for 0, 2, 4 and 6 days. The data represents the mean ± SD of n = 3 independent experiments.

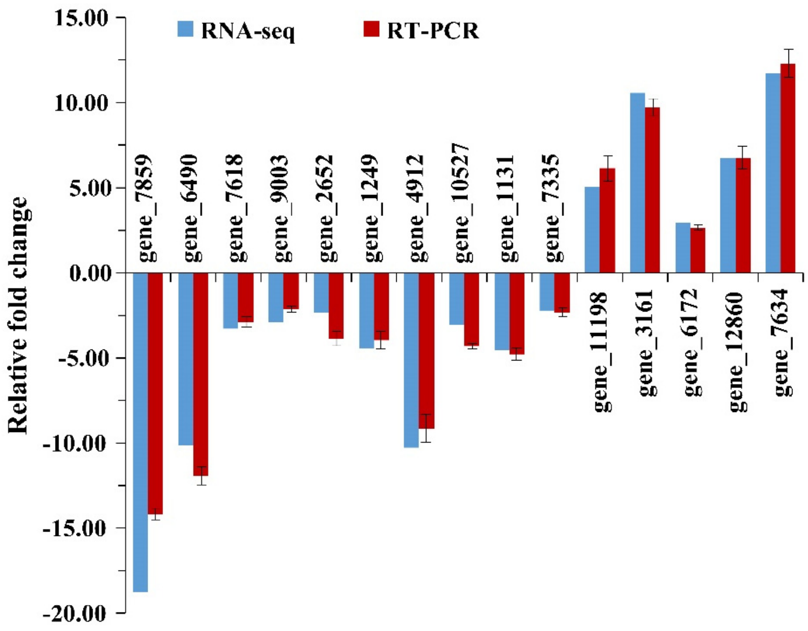

Figure S3. Gene expression levels of differentially expressed genes both in the real time PCR and RNA-seq data.

Supplementary Table S1. DEGs in chloroplast biogenesis and development

Gene ID	Log2 (Fold Change)	P-adj	Description
**Porphyrin and chlorophyll metabolism**			
gene_2801	-4.4227	7.17E-10	Protoporphyrinogen oxidase
gene_9773	-3.1681	1.06E-77	Magnesium-protoporphyrin IX monomethyl ester cyclase 2
gene_2957	-2.9920	1.80E-08	Uroporphyrinogen-III synthase
gene_11116	-2.9491	2.11E-64	Geranylgeranyl diphosphate reductase
gene_8958	-2.4772	2.18E-12	Chlorophyll synthase, chloroplastic
gene_12741	-2.3373	4.79E-26	Protochlorophyllide reductase, chloroplastic
gene_719	-2.2978	1.60E-06	Uroporphyrinogen decarboxylase 2
gene_12746	-2.2531	4.57E-37	Magnesium protoporphyrin IX methyltransferase
gene_10667	-2.2465	2.18E-26	Porphobilinogen deaminase, chloroplastic
gene_1131	-2.1790	6.49E-06	Chlorophyllide a oxygenase, chloroplastic
gene_3218	-2.1215	1.28E-03	Short chain dehydrogenase
gene_4997	-1.9147	3.66E-10	Glutamate--tRNA ligase, chloroplastic
gene_6037	-1.8764	5.06E-43	Delta-aminolevulinic acid dehydratase
gene_11931	-1.7299	1.90E-33	Divinyl chlorophyllide a 8-vinyl-reductase
gene_631	-1.4639	4.30E-22	Glutamate--tRNA ligase, cytoplasmic
gene_3787	-1.4070	2.60E-07	Magnesium-chelatase subunit ChlD
gene_6919	-1.3767	3.03E-11	Multicopper oxidase
gene_12658	-1.0722	7.56E-06	Glutamate-1-semialdehyde 2,1-aminomutase
gene_7319	1.1269	1.35E-06	Multicopper oxidase
gene_5475	1.1479	2.17E-05	Sirohydrochlorin cobaltochelatase
gene_348	1.8272	1.45E-29	Ferrochelatase-2, chloroplastic
gene_756	2.6255	3.28E-95	Tetrapyrrole (Corrin/Porphyrin) methylases
gene_4722	2.7587	7.93E-05	Protein NON-YELLOW COLORING 1-LIKE
gene_3161	3.4004	1.03E-06	Protein STAY-GREEN, chloroplastic
gene_11101	4.5913	3.00E-02	Protein NON-YELLOW COLORING 1-LIKE

**Thylakoid**			
gene_7859	-4.2295	9.77E-152	Photosystem I reaction center subunit III
gene_11272	-4.5658	4.56E-22	Photosystem II reaction center X protein (PsbX)
gene_6838	-4.5498	3.77E-130	Oxygen evolving enhancer protein 3 (PsbQ)
gene_1173	-4.4406	1.03E-139	Photosystem I reaction center subunit XI
gene_5065	-4.0623	1.11E-82	Photosystem II reaction center W protein
gene_989	-4.0112	1.14E-11	Photosystem I reaction center subunit N
gene_6360	-3.9490	7.98E-111	Photosystem I reaction center subunit V
gene_3326	-3.8987	7.44E-14	Oxygen-evolving enhancer protein 2
gene_12768	-3.8522	1.31E-22	Oxygen-evolving enhancer protein 1
gene_10589	-3.7829	4.68E-63	Oxygen-evolving enhancer protein 2
gene_7696	-3.7137	1.34E-02	Photosystem I reaction center subunit VI
gene_10201	-3.6719	8.39E-118	Photosystem I reaction center subunit IV
gene_5865	-3.4432	6.71E-122	Photosystem I reaction center subunit psaK
gene_9453	-3.3892	4.31E-17	PsbP-related thylakoid lumenal protein 2
gene_4661	-3.1879	9.23E-73	Photosystem II 10 kDa polypeptide PsbR
gene_4149	-2.4831	8.98E-74	Photosystem I 20 kDa subunit
gene_137	-2.0878	4.76E-04	PsbP-related thylakoid lumenal protein 4
gene_2652	-1.2397	1.29E-21	Cytochrome b6-F complex Fe-S subunit

Supplementary Table S2. Differentially expressed genes in fatty acid and protein metabolism

Gene ID	Log_2 (Fold Change)	P-adj	Description
**Fatty acid degradation**			
Gene_1965	-3.3029	1.07E-49	Betaine aldehyde dehydrogenase 2
Gene_9717	-2.8614	3.17E-53	Aldehyde dehydrogenase family 2 member B4
Gene_6171	1.3526	1.08E-18	Acyl-CoA dehydrogenase
Gene_2645	1.4552	2.28E-29	Triacylglycerol lipase SDP1
Gene_6172	1.5748	1.15E-22	Acyl-CoA oxidase
Gene_2888	1.6523	1.34E-33	Triacylglycerol lipase SDP1
Gene_5997	1.7628	2.09E-51	Acetyl-CoA acyltransferase 2
Gene_445	1.9168	3.30E-59	Acyl-CoA oxidase
Gene_8308	2.1108	8.17E-13	Enoyl-CoA hydratase/isomerase
Gene_8309	2.1542	3.79E-18	Enoyl-CoA delta isomerase 1
Gene_5055	2.3115	1.55E-51	Diglyceride acyltransferase 2
**Fatty acid biosynthesis**			
Gene_8382	-3.9788	2.06E-14	Enoyl-CoA reductase
Gene_4053	-3.4552	2.09E-30	3-oxoacyl-[acyl-carrier-protein] reductase
Gene_8174	-2.3126	7.45E-66	Malonyl CoA-acyl carrier protein transacylase
Gene_5277	-1.8903	1.53E-02	3-hydroxyacyl-[acyl-carrier-protein] dehydratase
Gene_11233	-1.7417	1.65E-02	Very-long-chain 3-oxoacyl-CoA reductase 1
Gene_12732	-1.7003	3.47E-07	3-oxoacyl-[acyl-carrier-protein] synthase I
Gene_12049	-1.6854	3.09E-03	3-oxoacyl-[acyl-carrier-protein] synthase II
Gene_4636	1.2566	1.92E-15	18:0-acyl-carrier protein thioesterase
**Ribosome**			
Gene_8933	-5.9784	2.77E-05	40S ribosomal protein S26
Gene_5202	-2.5299	2.08E-21	60S ribosomal protein L12
Gene_8822	-2.4225	1.61E-22	50S ribosomal protein L6
Gene_3979	-2.0513	4.89E-20	30S ribosomal protein S6
Gene_7314	-1.8234	2.31E-23	50S ribosomal protein L17

Gene_10132	-1.8013	8.87E-20	30S ribosomal protein S13
Gene_4374	-1.7643	6.83E-45	50S ribosomal protein L13
Gene_10527	-1.6196	8.40E-12	30S ribosomal protein S17
Gene_5152	-1.5302	7.96E-09	50S ribosomal protein L3
Gene_3679	-1.5244	1.85E-04	30S ribosomal protein S16
Gene_8776	-1.5125	1.47E-14	50S ribosomal protein L18
Gene_658	-1.4727	3.80E-26	50S ribosomal protein L4
Gene_7553	-1.4587	6.02E-05	30S ribosomal protein S9
Gene_9634	-1.4563	3.11E-10	30S ribosomal protein S5
Gene_2890	-1.4100	1.78E-16	50S ribosomal protein L31
Gene_5161	-1.3279	1.95E-24	30S ribosomal protein S10
Gene_10743	-1.3132	4.08E-30	50S ribosomal protein L3-1
Gene_7503	-1.2011	7.70E-06	50S ribosomal protein L22
Gene_8576	-1.1509	2.23E-03	50S ribosomal protein L4
Gene_5992	-1.1229	1.59E-06	50S ribosomal protein L9
Gene_8281	-1.0852	6.75E-06	30S ribosomal protein S5
Gene_9706	-1.0705	4.63E-02	50S ribosomal protein L28
Gene_11466	-1.0450	1.22E-06	50S ribosomal protein L33
Gene_8262	-1.0448	2.16E-11	50S ribosomal protein L28
Gene_8946	-1.0445	7.67E-12	50S ribosomal protein L15
Gene_4316	1.0237	2.78E-22	40S ribosomal protein S27
Gene_12772	1.0462	6.58E-24	60S ribosomal protein L10
Gene_9899	1.1199	3.68E-12	40S ribosomal protein S7
Gene_7476	1.1504	9.12E-28	40S ribosomal protein S2
Gene_6837	1.3338	1.06E-36	60S ribosomal protein L4
Gene_6732	1.4195	4.35E-17	50S ribosomal protein L2
Gene_11209	1.4338	3.14E-36	50S ribosomal protein L24
Gene_8169	2.2370	6.60E-05	50S ribosomal protein L13

Supplementary Table S3. Primers used for identification and RT-PCR in this study

**Primer name**	**Primer sequence (5′-3′)**
18S rDNA F	CACCTGGTTGATCCTGCCAG
18S rDNA R	AGCTTGATCCTTCTGCAGGTTCACCTAC
ITS F	ATGCGATACTTGGTGTGAAT
ITS R	GACGCTTCTCCAGACTACAAT
Gene_7859 F	CCCCACCATCGGCTTCATC
Gene_7859 R	CCATCAGGCTGCCGTTCTTGT
Gene_6490 F	CTCCAACAGCAGCCTCAACTCC
Gene_6490 R	CCGCCCACGATGTCAAAGC
Gene_7618 F	GGGTGTTTGCTTTGGCGTTCC
Gene_7618 R	CAGCCTTGCGGTCTGGGTAG
Gene_9003 F	GCCTTTCCTCCGGCGGTTTG
Gene_9003 R	TGGCCTCAGCCAGCATTCC
Gene_2652 F	GCGACGCCACCTACCTGATTG
Gene_2652 R	GAACTTGTTCTCGGCCTTGTTCC
Gene_1249 F	GCTGGTTGCATAACCCTGTCC
Gene_1249 R	GAGGCTGCGTTGTAGTTGTCG
Gene_4912 F	CCCCGCCTACCTGGAGAACC
Gene_4912 R	GGCCGTGGAACACCTCAGACT
Gene_10527 F	AAGACGGCGGTGGTGGAGGT

Gene_10527 R	GCTCGTCGTGCGCCATGTAC
Gene_1131 F	CCCCAGGAGTGCCAGAACCA
Gene_1131 R	AGTCGGTGGACATTCGGTAGAGC
Gene_7335 F	CTTACTCTGGCGGCGTGTT
Gene_7335 R	CCCTGGCTGTTGATGTTGG
Gene_11198 F	AGCGGGCAGCAACTGACAAG
Gene_11198 R	CAGGTGCAGCACAGCATAAAGAA
Gene_3161 F	CGACGAGGTGCTGGCTGAGTG
Gene_3161 R	AAGCGGTGCGTGCCAGGTT
Gene_6172 F	ACACCACCGCCTACGTCAACTC
Gene_6172 R	CGCCCTCAAAGGTCTGGAAGA
Gene_12860 F	TTTGGCGTGACAATGACTTCCC
Gene_12860 R	CGCAATCTGCTGCTCTACCTCC
Gene_7634 F	AACCGCATTTACCGAGACCTGAA
Gene_7634 R	CAGCGTGAGCGTGAGCACCA
Gene_5878 F	GGCTCCATCCTGTCCTCCCT
Gene_5878 R	CACATTGTGCCCGACCCAC
